# Central Adaptation to Repeated Galvanic Vestibular Stimulation: Implications for Pre-Flight Astronaut Training

**DOI:** 10.1371/journal.pone.0112131

**Published:** 2014-11-19

**Authors:** Valentina Dilda, Tiffany R. Morris, Don A. Yungher, Hamish G. MacDougall, Steven T. Moore

**Affiliations:** 1 Human Aerospace Laboratory, Department of Neurology, Icahn School of Medicine at Mount Sinai, New York, New York, United States of America; 2 School of Psychology, University of Sydney, Sydney, Australia; University of Otago, New Zealand

## Abstract

Healthy subjects (N = 10) were exposed to 10-min cumulative pseudorandom bilateral bipolar Galvanic vestibular stimulation (GVS) on a weekly basis for 12 weeks (120 min total exposure). During each trial subjects performed computerized dynamic posturography and eye movements were measured using digital video-oculography. Follow up tests were conducted 6 weeks and 6 months after the 12-week adaptation period. Postural performance was significantly impaired during GVS at first exposure, but recovered to baseline over a period of 7–8 weeks (70–80 min GVS exposure). This postural recovery was maintained 6 months after adaptation. In contrast, the roll vestibulo-ocular reflex response to GVS was not attenuated by repeated exposure. This suggests that GVS adaptation did not occur at the vestibular end-organs or involve changes in low-level (brainstem-mediated) vestibulo-ocular or vestibulo-spinal reflexes. Faced with unreliable vestibular input, the cerebellum reweighted sensory input to emphasize veridical extra-vestibular information, such as somatosensation, vision and visceral stretch receptors, to regain postural function. After a period of recovery subjects exhibited dual adaption and the ability to rapidly switch between the perturbed (GVS) and natural vestibular state for up to 6 months.

## Introduction

A non-physiological means of generating afferent vestibular input in the absence of head motion is the application of low amplitude (typically <5 mA) low frequency (<1 Hz) electrical currents between surface electrodes over the mastoid processes, termed bilateral bipolar Galvanic vestibular stimulation (GVS). GVS bypasses the mechanotransduction mechanism of hair cells of the vestibular labyrinth and acts at the junction between hair cells and the primary afferents (spike trigger zone); cathodal currents depolarize the trigger site and lead to excitation, whereas anodal currents hyperpolarize resulting in inhibition [1]. GVS activates neurons from both the otoliths and semicircular canals [1], [2], with all susceptible (predominantly irregular [1]–[4]) afferents triggered regardless of the directional preference of the hair cells they innervate [1], [5]. Thus, the pattern of activation by GVS is unlike any produced by natural motion, with canal and otolith afferents of all directional sensitivities being activated by cathodal stimulation (or inhibited by anodal current) simultaneously.

An intact vestibular nerve is required to generate a response to GVS [5] (with no evidence of direct vestibular nuclei or cerebellar activation [6]), and human fMRI studies have demonstrated that CNS activation in response to GVS reflects that of natural (motion-initiated) vestibular afferent signals [5]. There is considerable perceptual, postural and ocular data indicating otolith-mediated responses to GVS [7]–[14]). Our own oculomotor data shows primarily otolith (roll position, upper pole of the eye towards the anode) plus a small canal response (yaw and roll nystagmus <5°/s, with slow phase velocity towards the anode [15], [16]), entrained to a 5 mA peak pseudorandom GVS waveform. GVS application when quietly standing results in a vestibulo-spinal reflex response [9], [17]–[19] manifested as a small (7 mm RMS for 5 mA peak pseudorandom GVS [9]) mediolateral displacement of the center of mass towards the anode; subjectively, the perception is of an unstable surface rather than self-motion, with the ground ‘rocking’ slightly from side to side in a random manner, as if on a boat in rough waters [20]. These results are consistent with the notion that the CNS interprets the resultant sum of all vestibular afferents activated by GVS as a head tilt in the direction of the cathode [5], and generates small reflex responses towards the anode. A previous study suggests that these reflex responses do not adapt to repeated GVS exposure [21].

Our studies have shown that anteroposterior stability (cerebellum) [9], obstacle course navigation (cortex/cerebellum) [11], and fine motor control (cortex/cerebellum) [20], as well as short-term spatial memory (hippocampus), perspective taking and perception of motion (cortex) [20], [22], are degraded by bilateral bipolar GVS. These findings suggest that imposing pseudorandom GVS ‘noise’ on veridical vestibular input at the spike trigger zone negatively impacts upstream central functions that rely on integrated vestibular, visual and somatosensory input. We have leveraged this destabilizing effect of GVS on sensorimotor function to replicate in healthy subjects the decrements in postural [9], locomotor [11], oculomotor [11], and fine motor [20] performance observed in astronauts after spaceflight.

The aim of the current study was to determine if repeated exposure to a pseudorandom (sum of sines) GVS waveform over an extended period promoted central adaptation, with recovery of complex sensorimotor function during Galvanic vestibular stimulation. In addition, we wished to ascertain whether low-level vestibular reflex responses, such as the vestibulo-ocular reflex, were unaffected by repeated GVS application, as suggested by a previous study [21]. Recovery of centrally-mediated motor function in the presence of unpredictable vestibular ‘noise’ may provide a training paradigm to ‘pre-habilitate’ individuals (such as astronauts or vestibular patients about to undergo planned unilateral deafferentiation) to novel provocative vestibular environments.

## Methods

### Ethics Statement

Ten healthy subjects, 7 males/3 females, with a mean age of 26.1 yrs (SD 2.0), participated in this study. The Program for the Protection of Human Subjects (PPHS) at Icahn School of Medicine at Mount Sinai approved the experiments (study 07-0468), and subjects gave their written informed consent and were free to withdraw at any time.

### Galvanic Vestibular Stimulation (GVS)

The pseudorandom bilateral-bipolar Galvanic stimulus consisted of a sum-of-sines (0.16, 0.33, 0.43, 0.61 Hz) with peak amplitude of 5 mA [9], [11]. An optically-isolated constant current generator delivered the current to the surface of the subject's skin via leads and large electrodes placed over the mastoid processes, cut from electrosurgical split grounding plate electrodes (7180, 3M Health Care, St. Paul, MN). The electrodes were coated with an additional layer of EMG electrode gel then applied to the surface of the subject's skin using the electrode's adhesive surround, and a piece of insulated tape was added to the skin underneath the bare metal tag. A soft pad was placed over each electrode and held firmly in place by an elasticized strap. The electrodes and strap did not produce discomfort or restrict head movement. Prior to testing subjects were briefly exposed to the Galvanic stimulus to ensure that there was no adverse cutaneous effects. We have previously demonstrated that the GVS analog is well tolerated in the vast majority of subjects during extended exposure (up to 20 min) at amplitudes up to 5 mA [23].

### Computerized Dynamic Posturography (CDP)

**Figure 1:**
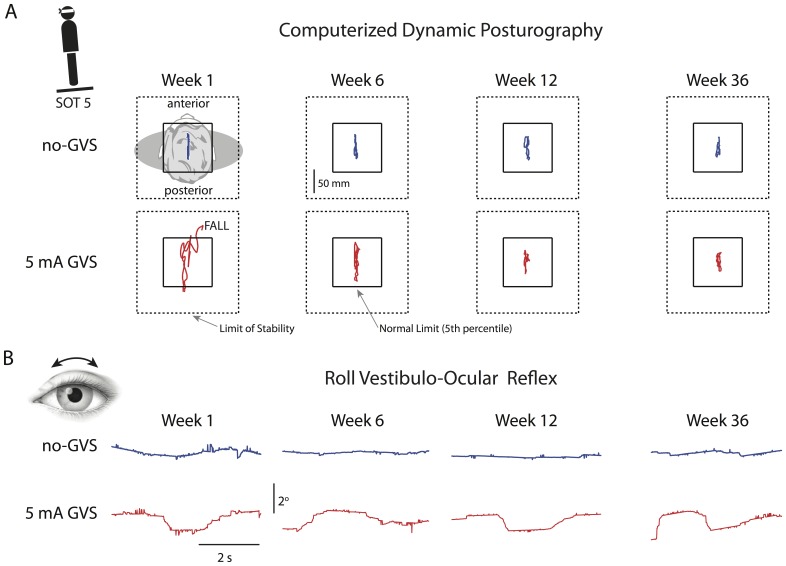
Posturography and ocular torsion data from a typical subject. (A) Anterioposterior sway plots without (upper row blue trace) and with (lower row red trace) 5 mA GVS for SOT 5 (eyes closed and sway-referenced support surface). The subject fell during initial exposure to GVS, but anterioposterior sway decreased to baseline levels over the 12 weeks of GVS exposure, and this recovery was maintained 6 months after adaption. (B) Torsional (roll) eye position traces without (upper row blue trace) and with (lower row red trace) 5 mA GVS. In contrast to the posturography data, the vestibulo-ocular reflex response to GVS was unchanged by repeated GVS exposures.

The standardized CDP battery (Equitest, Neurocom, Clackamas OR) comprises six sensory organization tests (SOTs) to evaluate the relative contribution and effectiveness of vision, somatosensory (proprioception, primarily from the ankles) and vestibular input to the CNS to maintain postural stability [24]. This is achieved by selectively degrading visual (closing the eyes or sway-referencing of the visual surround in the anteroposterior direction) and proprioceptive input (sway-referencing of the support surface in the anteroposterior direction). The six conditions are: *SOT 1* eyes open, stable support; *SOT 2* eyes closed, stable support; *SOT 3* eyes open + sway-referenced visual surround, stable support; *SOT 4* eyes open, sway-referenced support; *SOT 5* eyes closed, sway-referenced support; *SOT 6* eyes open + sway-referenced visual surround and support. Subjects performed 3 trials of each of the 6 SOTs without GVS as a baseline, then repeated the 18 trials with GVS at peak amplitude of 5 mA. For each trial the device calculates an equilibrium score based on the peak anteroposterior sway amplitude as a percentage of the theoretical limits of stability (Fig. 1A; 12.5° peak to peak [24]; for a detailed analysis see [9]). An equilibrium score of 100 indicates no sway, a value of zero denotes sway beyond the limits of stability or a fall. The composite equilibrium score is a weighted average of all trials. Sensory indices were calculated using a ratio of the equilibrium score from an SOT that primarily invokes one of the three sensory modalities (somatosensory - SOT 2; visual - SOT 4; vestibular - SOT 5) relative to the SOT 1 baseline (eyes open stable support) [24].

### Roll Eye Movement Response

The ocular roll response was assessed both with and without GVS utilizing a device developed by the investigators [25], [26]. The eye movement response to mastoidal surface GVS occurs primarily in roll (around the line of sight). The largest response is generated by sustained direct current stimulation, diminishing with increasing GVS frequency [10], [16], [27], [28]. In the current study, roll eye movements were primarily in response to the lowest (0.16 Hz) GVS frequency component (see Fig. 1B), consistent with an otolith-ocular tilt reflex as described in the Introduction. Small spontaneous roll eye movements are commonly observed in the absence of GVS or motion stimuli, particularly in darkness [29], [30]. Little is known about the frequency characteristics of these movements, which typically manifest as a slow drift in roll eye position with amplitudes of up to 1° (see Fig. 1B).

Subjects were seated and wore tight-fitting light occluding goggles with firewire digital video cameras attached. With the subject looking straight ahead in darkness, 60 s of binocular monochrome digital images were acquired at a rate of 60 Hz and saved to disk for later analysis, for both the no-GVS and 5 mA GVS condition. Epochs of at least 10 s of video in which no blinks or other artifacts were present were utilized to calculate torsional (roll) eye position. The pupil center was determined with a center of mass algorithm [25], [26], [30]. Pixels within the iris were then sampled using elliptical annuli centered on the pupil and cross-correlation of these signals provided the amount of relative rotation about the line of sight between two images [26], [30], [31]. These image processing algorithms have demonstrated an accuracy and resolution of the order of 0.1° [26]. The maximum and minimum amplitude of the eye movement data was used to calculate the peak-to-peak roll amplitude within each epoch.

### Experimental Protocol

Subjects were tested at weekly intervals for 12 weeks, returning for follow-up sessions six weeks (week 18) and six months (week 36) after the final adaptation session (week 12). In each session subjects were exposed to a cumulative exposure of 10 minutes of pseudorandom, 5 mA peak, bilateral bipolar GVS. Subjects initially performed posturography testing without GVS, then repeated the computerized dynamic posturography assessment with GVS (total GVS exposure 6 min). A further 60 s of GVS exposure occurred during the roll vestibulo-ocular reflex testing, and the balance of GVS exposure (3 min) was delivered while subjects walked about the hallways surrounding the laboratory, including tandem (heel-toe) walking.

### Statistical Analysis

The postural and ocular response to GVS over the 14 test sessions was assessed with a two-way repeated measures analysis of variance (ANOVA). Main factors were GVS condition (no GVS, 5 mA GVS) and test session (1 through 14). Follow-up analysis was conducted with a paired two-tailed t-test.

## Results

Posturography data from an individual subject (Fig. 1A) illustrate central adaptation to GVS over the 12 weekly exposures and retention at the 6-month follow-up. During computerized dynamic posturography without GVS anterioposterior sway was essentially unchanged over 36 weeks. When first exposed to GVS (week 1) body sway exceeded clinical norms and the subject fell when the vestibular system was challenged (SOT 5: eyes closed and sway-referenced support). An improvement in anterioposterior sway during GVS was apparent by week 6, although the extent of sway was considerably larger than in the no-GVS baseline condition. After 12 weekly GVS exposures (120 min total) body sway during GVS was considerably reduced and comparable to the baseline no-GVS data, and this recovery was retained when exposed to GVS 6 months later. In contrast, the roll vestibulo-ocular reflex response to GVS (Fig. 1B) was unchanged over the 12 weekly GVS exposures and at the 6-month follow-up.

**Figure 2:**
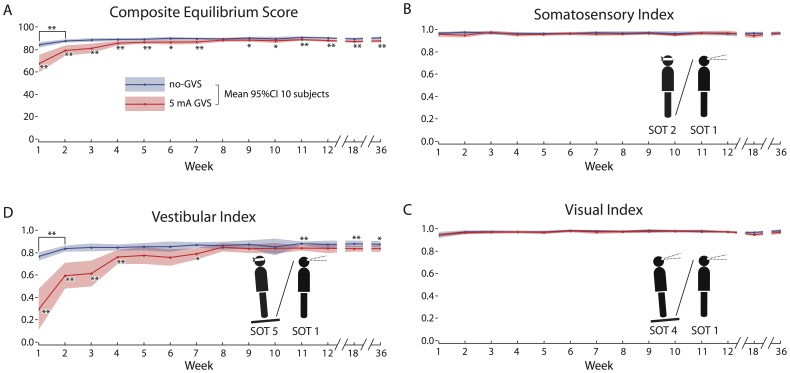
Computerized dynamic posturography data from all 10 subjects (mean and 95%CI) without (blue trace) and with (red trace) 5 mA GVS. (A) The composite equilibrium score was significantly lower when first exposed to GVS, but recovered to baseline after 7 weeks (70 min total) exposure, and this recovery was retained for up to 6 months post adaptation. (B) The somatosensory index, and (C) the visual index, were unaffected by GVS exposure. (D) In contrast, the vestibular index (SOT5/SOT1 ratio), was significantly affected by GVS exposure, illustrating the almost exclusive impact of GVS on the vestibular component of postural control. The vestibular index recovered to baseline by week 8, following 70 min of cumulative GVS exposure, and this recovery was maintained 6 months post-adaptation.

This pattern was consistent across the 10 subjects. The means and standard deviations for the no-GVS and GVS conditions at each test session for composite CDP score and vestibular index are presented in Table 1, and raw CDP data are available in Data S2. The two-factor repeated measures ANOVA showed significant effects on CDP composite score (Fig. 2A) of GVS condition F(1,13) = 51.9, p = 0.00005, test session F(1,13) = 29.9, p = 0.0004, and GVS condition/test session interaction F(1, 13) = 21.8, p = 0.001. In the no-GVS condition the effect of test session was entirely due to a small but significant (t[9] = 3.64; p = 0.0054) 4% increase in the composite score from week 1 to week 2 (Fig. 2A; blue trace), most likely the previously noted practice effect [24]. The effect of test session was significantly stronger for the 5 mA GVS condition. The CDP composite score was significantly lower than the no-GVS baseline for weeks 1 through 7 (Table 1), but by the 8th week (cumulative GVS exposure of 80 minutes) computerized dynamic posturography performance had returned to within 3% of baseline, and this recovery persisted for the remainder of the weekly sessions and at the 6 week and 6 month follow ups (Fig. 2A; red trace).

**Table 1:** Comparison of no-GVS vs GVS Conditions (paired t-test, df = 9, two-tailed).

	CDP Composite EQ Score			CDP Vestibular Index			Ocular Torsion (deg)		
Session	No GVS	GVS	p	No GVS	GVS	p	No GVS	GVS	p
Week 1	84.0 (4.0)	67.2 (12)	0.001*	76.6 (6.0)	29.4 (30)	0.001*	0.86 (0.7)	2.4 (1.3)	0.005*
Week 2	87.4 (2.3)	78.9 (7.5)	0.004*	83.6 (4.5)	59.4 (19)	0.002*	0.53 (0.4)	2.3 (1.6)	0.006*
Week 3	88.3 (2.7)	80.8 (6.9)	0.001*	84.7 (5.7)	61.4 (19)	0.004*	0.55 (0.3)	2.4 (1.7)	0.001*
Week 4	88.8 (1.8)	85.5 (3.4)	0.003*	84.6 (4.5)	76.1 (10)	0.004*	0.84 (0.9)	1.9 (1.4)	0.077
Week 5	88.9 (2.0)	86.3 (2.4)	0.007*	85.3 (5.5)	77.5 (12)	0.093	0.86 (0.7)	2.0 (1.4)	0.039*
Week 6	89.7 (2.4)	86.4 (3.5)	0.021*	85.3 (7.5)	75.7 (12)	0.078	1.19 (1.0)	2.3 (1,5)	0.062
Week 7	89.5 (1.4)	86.6 (3.2)	0.002*	86.9 (3.8)	78.9 (8.2)	0.003*	0.79 (0.6)	2.1 (1.8)	0.049*
Week 8	89.3 (1.6)	88.1 (2.4)	0.058	86.2 (5.8)	84.8 (6.1)	0.398	0.54 (0.3)	1.6 (1.2)	0.020*
Week 9	90.0 (1.8)	88.1 (2.2)	0.020*	87.4 (5.9)	83.5 (6.2)	0.135	0.72 (0.6)	2.0 (1.5)	0.028*
Week 10	89.3 (3.1)	87.5 (3.1)	0.048*	85.1 (12)	83.9 (7.7)	0.765	1.08 (0.8)	1.7 (1.3)	0.230
Week 11	90.5 (1.6)	88.6 (1.5)	0.004*	88.1 (2.9)	84.1 (3.7)	0.009*	0.96 (0.9)	2.2 (1.7)	0.062
Week 12	90.1 (1.2)	87.8 (2.0)	0.005*	87.3 (5.6)	83.9 (7.0)	0.265	0.68 (0.4)	2.7 (1.8)	0.007*
Week 18	89.2 (1.8)	86.9 (1.9)	0.007*	87.5 (4.7)	83.5 (4.1)	0.001*	0.87 (0.6)	2.4 (1.7)	0.019*
Week 36	90.2 (1.9)	87.5 (2.4)	0.001*	87.5 (4.4)	83.6 (4.9)	0.030*	1.40 (0.8)	2.6 (1.7)	0.064

Comparison of mean (SD) computerized dynamic posturography (CDP) composite equilibrium (EQ) score and vestibular index, and peak-to-peak ocular torsion, for each of the 14 test sessions over 36 weeks.

The decrements in computerized dynamic posturography performance during GVS were solely due to a degradation of vestibular input to postural control. There was no effect of GVS or test session on the somatosensory index (GVS condition F(1,13) = 3.5, p = 0.10, test session F(1,13) = 0.0003, p = 0.98, and GVS condition/test session interaction F(1,13) = 0.13, p = 0.72; Fig. 2B) or visual index (GVS condition F(1,13) = 2.9, p = 0.12, test session F(1,13) = 5.2, p = 0.05, and GVS condition/test session interaction F(1,13) = 0.59, p = 0.46; Fig. 2C). There was a highly significant effect of GVS condition and test session on the vestibular index (Fig. 2D), the ratio of equilibrium scores on SOT 5 (eyes closed and sway-referenced support) and the SOT 1 baseline (eyes open fixed support); GVS condition F(1,13) = 93.0, p = 0.000005, test session F(1,13) = 53.2, p = 0.00005, and GVS condition/test session interaction F(1,13) = 33.6, p = 0.0003. Consistent with the composite score results above, in the no-GVS condition the effect of test session was due to a practice effect. There was a significant (t[9] = 6.7; p = 0.0001) 9% increase in the vestibular index from week 1 to week 2, which remained stable over the ensuing 35 weeks. The vestibular index exhibited a marked 62% decrease on week 1 during GVS exposure that recovered to the no-GVS baseline by week 8 (Table 1), and recovery was maintained throughout further testing out to 36 weeks (Fig. 2D).

**Figure 3:**
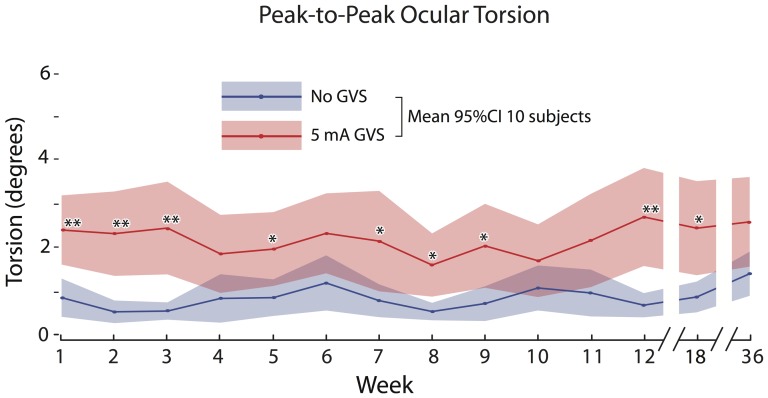
Torsional (roll) eye position data from all 10 subjects (mean and 95%CI) without (blue trace) and with (red trace) 5 mA GVS. There was a significant increase in ocular torsion during GVS relative to baseline, which was unaffected by repeated GVS exposure.

In contrast to the posturography results, there was no effect of test session (F(1,13) = 0.08, p = 0.21) on the ocular roll response (see Table 1; ocular roll data are available in Data S2). As established previously [10], [16], [27], [28], there was a highly significant effect of GVS condition, with an overall mean peak-to-peak roll during GVS of 2.19° [CI 0.26], which was significantly larger (t[139] = 41.6; p<<0.0001; see table 1 for mean and SD values) than the baseline no-GVS average of 0.85° [CI 0.11]. Mean ocular torsion per weekly test was significantly larger during GVS than baseline for 9 of the 14 sessions (Fig. 3; Table 1).

## Discussion

The results of this study demonstrate adaptation to repeated externally applied pseudorandom Galvanic stimulation of the vestibular labyrinths. Subject performance on a computerized dynamic posturography task was significantly impaired during GVS at first exposure, but recovered to baseline over a period of 7–8 weeks (70–80 min exposure). This recovery of postural performance was maintained at 6 weeks and 6 months after the 12-week period of adaptation. The roll eye movement response to GVS, which most likely reflects a linear ocular ‘counter roll’ response to low-frequency (0.16 Hz) otolith stimulation [7]–[14]), was not attenuated by repeated GVS exposure.

Direct reflex responses to GVS, such as ocular torsion, were unchanged over the 12-week period of exposure to GVS (and at follow-up). Subjects experienced the rolling of the visual field and illusory sensations of tilt throughout the 36 weeks of testing. In contrast, complex behavior that required central integration of vestibular, visual and somatosensory input at the level of the cerebellum, such as posturography and heel-toe walking (see Video S1) recovered to baseline following adaptation to repeat GVS exposures. For example, at week 1 and week 12 the ocular roll response to GVS was unchanged. This suggests that during posturography the afferent vestibular input to the cerebellum at these time points was similarly degraded by unpredictable GVS ‘noise’, the amplitude of which was considerably larger than veridical vestibular input from the subjects' own motion [9]. However, postural performance at week 12 had fully recovered to the no-GVS baseline. This suggests that GVS adaptation did not occur at the vestibular end-organs or involve changes in low-level (brainstem-mediated) vestibulo-ocular or vestibulo-spinal reflexes. We speculate that faced with unreliable vestibular input, the cerebellum reweighted sensory input to emphasize veridical extra-vestibular information, such as somatosensation, vision and visceral stretch receptors, to regain postural and locomotor function during GVS exposure. The fact that postural performance during GVS was maintained up to 6 months post-adaptation suggests that subjects retained this sensory reweighting and could switch cerebellar states (dual adaptation) depending on the vestibular context (GVS or natural vestibular conditions). There were no adverse effects on baseline postural performance; in fact, computerized dynamic posturography scores improved slightly when tested after GVS exposure [9].

A similar adaptation may occur in astronauts during extended weightlessness. In-flight data suggests central reorganization of sensory input, up-weighting vision, proprioception and visceral input [32]–[34], to compensate for the lack of low-frequency otolith input and unloading of visceral stretch receptors. This sensory reorganization is a necessary and positive process, facilitating life in space much as the baseline gravitational state facilitates life on Earth. Problems arise from the time required to switch between these two sensorimotor states. The post-flight effects are better documented; impaired balance [35]; gait [36]; motion perception [37], and piloting performance [20], [38] are common for up to 2 weeks post-landing. Veteran astronauts maintain critical central elements of their microgravity-adapted state, as indicated by a significant improvement in motion sickness symptoms [39] and post-flight sensorimotor performance [35], [36] on subsequent missions. Analogous to our GVS-adapted subjects, veteran crewmembers exhibit dual adaptation to a perturbed (microgravity) and natural vestibular environment, and the ability to retain and rapidly switch between these states at the critical inertial transitions of orbital insertion into microgravity and return to terrestrial gravity.

The vestibular perturbation of space flight and GVS are clearly different; the almost total lack of DC linear acceleration in microgravity and low-frequency pseudorandom ‘noise’ superimposed on afferent vestibular input by GVS. Common to both conditions, however, is the primary effect on low frequency otolith-mediated responses, a discord between afferent angular and linear information signaling head movement that is outside the realm of the physically possible in a terrestrial environment, and an adaptive sensory reweighting resulting in dual-adapted (perturbed/normal) sensorimotor states. Moreover, acute exposure to GVS accurately replicates post-flight postural, locomotor, perceptual, and fine motor impairment due to microgravity adaptation [9], [11], [20], [22]. Pre-flight adaptation to GVS may provide some level of protection when exposed to the novel inertial environments of spaceflight, which in this study persisted at least as long as a typical mission to the International Space Station (6 months). In a similar manner, GVS adaptation may be useful in vestibular ‘pre-habilitation’ [40], in which patients with planned unilateral deafferentiation are exposed to repeated provocative vestibular stimuli prior to intervention to minimize the impact of a post-lesion imbalance in vestibular tone.

## Supporting Information

Data S1 **Computerized posturography data (equilibrium scores and sensory indices).**
(XLSX)

Data S2 **Ocular torsion (roll) position data in degrees.**
(XLSX)

Video S1 **A subject performing heel-toe (tandem) walking at week 1 and week 12 while experiencing 5 mA GVS.** Note the recovery of locomotor function at week 12 following 120 min of cumulative GVS exposure.
(AVI)

## References

[1] Goldberg JM, Smith CE, Fernandez C (1984) Relation between discharge regularity and responses to externally applied galvanic currents in vestibular nerve afferents of the squirrel monkey. Journal of neurophysiology 51: 1236–1256. 6737029 10.1152/jn.1984.51.6.1236

[2] Kim J, Curthoys IS (2004) Responses of primary vestibular afferents to galvanic vestibular stimulation (GVS) in the anaesthetised guinea pig. Brain Res Bull 64: 265–271. 15464864 10.1016/j.brainresbull.2004.07.008

[3] Kleine JF, Guldin WO, Clarke AH (1999) Variable otolith contribution to the galvanically induced vestibulo-ocular reflex. Neuroreport 10: 1143–1148. 10321499 10.1097/00001756-199904060-00044

[4] McCrea R, Gdowski G, luan H (2001) Current concepts of vestibular nucleus function. Ann NY Acad Sci 942: 328–344. 11710475 10.1111/j.1749-6632.2001.tb03758.x

[5] Fitzpatrick RC, Day BL (2004) Probing the human vestibular system with galvanic stimulation. J Appl Physiol 96: 2301–2316. 15133017 10.1152/japplphysiol.00008.2004

[6] Cass SP, Redfern MS, Furman JM, DiPasquale JJ (1996) Galvanic-induced postural movements as a test of vestibular function in humans. Laryngoscope 106: 423–430. 8614216 10.1097/00005537-199604000-00007

[7] Holstein GR, Friedrich VL Jr, Martinelli GP, Ogorodnikov D, Yakushin SB, et al (2012) Fos expression in neurons of the rat vestibulo-autonomic pathway activated by sinusoidal galvanic vestibular stimulation. Front Neurol 3: 4. 22403566 10.3389/fneur.2012.00004 PMC3289126

[8] Cohen B, Yakushin SB, Holstein GR (2011) What does galvanic vestibular stimulation actually activate? Front Neurol 2: 90. 22287951 10.3389/fneur.2011.00090 PMC3258665

[9] MacDougall H, Moore ST, Curthoys IS, Black FO (2006) Modeling postural instability with Galvanic vestibular stimulation. Exp Brain Res 172: 208–220. 16432695 10.1007/s00221-005-0329-y

[10] MacDougall HG, Brizuela AE, Burgess AM, Curthoys IS, Halmagyi GM (2005) Patient and normal three-dimensional eye-movement responses to maintained (DC) surface galvanic vestibular stimulation. Otol Neurotol 26: 500–511. 15891657 10.1097/01.mao.0000169766.08421.ef

[11] Moore ST, MacDougall H, Peters BT, Bloomberg JJ, Curthoys IS, et al (2006) Modeling locomotor dysfunction following spaceflight with Galvanic vestibular stimulation. Exp Brain Res 174: 647–659. 16763834 10.1007/s00221-006-0528-1

[12] Zink R, Steddin S, Weiss A, Brandt T, Dieterich M (1997) Galvanic vestibular stimulation in humans: effects on otolith function in roll. Neurosci Lett 232: 171–174. 9310307 10.1016/s0304-3940(97)00610-1

[13] Severac Cauquil A, Faldon M, Popov K, Day BL, Bronstein AM (2003) Short-latency eye movements evoked by near-threshold galvanic vestibular stimulation. Exp Brain Res 148: 414–418. 12541151 10.1007/s00221-002-1326-z

[14] Kim J (2012) Tonic eye movements induced by bilateral and unilateral galvanic vestibular stimulation (GVS) in guinea pigs. Brain Res Bull 10.1016/j.brainresbull.2012.09.010 23022577

[15] MacDougall H (2003) The human eye-movement response to maintained surface galvanic vestibular stimulation (PhD THESIS) [PhD]. Sydney: University of Sydney. 146 p.

[16] Curthoys IS, Macdougall HG (2012) What galvanic vestibular stimulation actually activates. Front Neurol 3: 117. 22833733 10.3389/fneur.2012.00117 PMC3400934

[17] Britton TC, Day BL, Brown P, Rothwell JC, Thompson PD, et al (1993) Postural electromyographic responses in the arm and leg following galvanic vestibular stimulation in man. Exp Brain Res 94: 143–151. 8335069 10.1007/BF00230477

[18] Day BL, Severac Cauquil A, Bartolomei L, Pastor MA, Lyon IN (1997) Human body-segment tilts induced by galvanic stimulation: a vestibularly driven balance protection mechanism. J Physiol 500 (Pt 3)661–672. 9161984 10.1113/jphysiol.1997.sp022051 PMC1159417

[19] Pavlik AE, Inglis JT, Lauk M, Oddsson L, Collins JJ (1999) The effects of stochastic galvanic vestibular stimulation on human postural sway. Exp Brain Res 124: 273–280. 9989432 10.1007/s002210050623

[20] Moore ST, Dilda V, MacDougall HG (2011) Galvanic vestibular stimulation as an analogue of spatial disorientation after spaceflight. Aviat Space Environ Med 82: 535–542. 21614868 10.3357/asem.2942.2011

[21] Guerraz M, Day BL (2001) Human body response to galvanic vestibular stimulation is not affected when the stimulus is self-triggered. J Physiol 531P: 142.

[22] Dilda V, MacDougall HG, Curthoys IS, Moore ST (2012) Effects of Galvanic vestibular stimulation on cognitive function. Exp Brain Res 216: 275–285. 22076407 10.1007/s00221-011-2929-z

[23] Dilda V, MacDougall HG, Moore ST (2011) Tolerance to Extended Galvanic Vestibular Stimulation: Optimal Exposure for Astronaut Training. Aviat Space Environ Med 82: 770–774. 21853854 10.3357/asem.3051.2011

[24] Nashner LM (1993) Computerized Dynamic Posturography. In: Jacobson GP, Newman CW, Kartush JM, editors. Handbook of Balance Function Testing. St Louis: Mosby Year Book. pp. 298–301.

[25] MacDougall HG, Moore ST (2005) Functional assessment of head-eye coordination during vehicle operation. Optom Vis Sci 82: 706–715. 16127336 10.1097/01.opx.0000175623.86611.03

[26] Moore ST, Haslwanter T, Curthoys IS, Smith ST (1996) A geometric basis for measurement of three-dimensional eye position using image processing. Vision Res Vol.36: 445–459. 10.1016/0042-6989(95)00130-1 8746234

[27] MacDougall HG, Brizuela AE, Burgess AM, Curthoys IS (2002) Between-subject variability and within-subject reliability of the human eye-movement response to bilateral galvanic (DC) vestibular stimulation. Exp Brain Res 144: 69–78. 11976760 10.1007/s00221-002-1038-4

[28] MacDougall HG, Brizuela AE, Curthoys IS (2003) Linearity, symmetry and additivity of the human eye-movement response to maintained unilateral and bilateral surface galvanic (DC) vestibular stimulation. Exp Brain Res 148: 166–175. 12520404 10.1007/s00221-002-1289-0

[29] Kori AA, Schmid-Priscoveanu A, Straumann D (2001) Vertical divergence and counterroll eye movements evoked by whole-body position steps about the roll axis of the head in humans. Journal of neurophysiology 85: 671–678. 11160502 10.1152/jn.2001.85.2.671

[30] Moore ST, McCoy SG, Curthoys IS (1991) VTM - an image processing system for measuring ocular torsion. Comp Meth Prog Biomed 35: 219–230. 10.1016/0169-2607(91)90124-c 1935015

[31] Haslwanter T, Moore ST (1995) A theoretical analysis of three dimensional eye position measurement using image processing. IEEE Trans BME BME-42 1053–1061. 10.1109/10.469371 7498908

[32] Clarke AH, Grigull J, Mueller R, Scherer H (2000) The three-dimensional vestibulo-ocular reflex during prolonged microgravity. Exp Brain Res 134: 322–334. 11045357 10.1007/s002210000476

[33] Clement G, Moore ST, Raphan T, Cohen B (2001) Perception of tilt (somatogravic illusion) in response to sustained linear acceleration during space flight. Exp Brain Res 138: 410–418. 11465738 10.1007/s002210100706

[34] Young LR, Mendoza JC, Groleau N, Wojcik PW (1996) Tactile influences on astronaut visual spatial orientation: human neurovestibular studies on SLS-2. J Appl Physiol 81: 44–49. 8828646 10.1152/jappl.1996.81.1.44

[35] Paloski WH, Reschke MF, Black FO (1999) Recovery of Postural Equilibrium Control Following Space Flight (DSO 605). In: Sawin CF, Taylor GR, Smith WL, editors. Extended Duration Orbiter Medical Project Final Report 1989–1995 (NASA/SP-1999-534). Houston: NASA. pp. 5.4: 1–16.

[36] Bloomberg JJ, Peters BT, Smith SL, Huebner WP, Reschke MF (1997) Locomotor head-trunk coordination strategies following space flight. J Vest Res 7: 161–177. 9178222

[37] Harm DL, Reschke MF, Parker DE (1999) Visual vestibular integration:motion perception reporting. In: Sawin CF, editor. Extended Duration Orbiter Medical Project. Houston: NASA Johnson Space Center. pp. 5.2-1-5.2-12.

[38] Moore ST, MacDougall HG, Lesceu X, Speyer JJ, Wuyts F, et al (2008) Head-eye coordination during simulated orbiter landing. Aviat Space Environ Med 79: 888–898. 18785358 10.3357/asem.2209.2008

[39] Davis JR, Vanderploeg JM, Santy PA, Jennings RT, Stewart DF (1988) Space motion sickness during 24 flights of the space shuttle. Aviat Space Environ Med 59: 1185–1189. 3240221

[40] Magnusson M, Karlberg M, Tjernstrom F (2011) ‘PREHAB’: Vestibular prehabilitation to ameliorate the effect of a sudden vestibular loss. NeuroRehabilitation 29: 153–156. 22027076 10.3233/NRE-2011-0689

